# Photobiomodulation therapy and the clinical reality in Brazil: response to the letter to the editor

**DOI:** 10.1186/s12891-020-03906-x

**Published:** 2021-01-10

**Authors:** Cid André Fidelis de Paula Gomes, Almir Vieira Dibai-Filho, Fabiano Politti, Adriano Rodrigues de Oliveira, Cheila de Souza Bacelar Pereira, Aron Charles Barbosa da Silva, Daniela Aparecida Biasotto-Gonzalez

**Affiliations:** 1 Postgraduate Program in Rehabilitation Sciences, Universidade Nove de Julho, Rua Vergueiro, 235/249, 2° Subsolo, Liberdade, São Paulo, SP CEP 01504-001 Brazil; 2 Postgraduate Program in Physical Education, Universidade Federal do Maranhão, São Luís, MA Brazil

We are extremely honored to know that our work has a wide reach within the area of photobiomodulation therapy. We understand the questions and conflicting points raised by the authors of the Letter to the Editor and consider them important. However, we consider that some caveats and explanations are necessary in response.

In terms of the disputes concerning the dose used in our research [1] and the study used as reference, we have used the study by Hegedus et al. [2] as the basis for defining the location and points for irradiation since the establishment of our first intervention protocol [3], as well as in another randomized clinical trial with a similar theme [2], which is already published.

Hegedus et al. [2] applied 6 J to each of the 8 points located on the knee of his volunteers. We adapt these same points and replicate those same Joules. It is quite true, using equipment with different characteristics. Our central idea, not only in relation to photobiomodulation therapy, is that a randomized clinical trial should reproduce normal clinical conditions, especially with respect to the use of therapeutic resources, in order to guarantee greater external validity of the results obtained. Thus, we selected photobiomodulation devices that are commonly used in clinical practice in Brazil.

In this way, we replicate doses and application points of a previously published study [2]; really using equipment with different and even inferior characteristics when compared to other studies. However, at no time do we hide the equipment’s characteristics. We assume this study limitation. In our country, most of the photobiomodulation devices regulated and available for clinical use by rehabilitation professionals do not present similar characteristics to those used in several other clinical studies already published, for example: non-compatibility for the use of clusters and low power equipment.

As previously highlighted, we prefer to use the reference of a double-blind, randomized, placebo-controlled trial [2]. Therefore, we chose not to replicate the recommendations of the World Association for Laser Therapy (WALT) [4]. In particular, we did not mention the highlighted meta-analysis [5], as we did not have access to it during the preparation and finalization of the manuscript. However, without a doubt it will be in our future manuscripts.

We also highlight that most of the researchers in our group have at least six other randomized controlled trials published [6–11], all of them with themes related to the effectiveness of therapeutic resources directed to musculoskeletal rehabilitation. From the experience acquired during these studies, we have adopted methodological processes that are judicious and necessary for the development of a randomized clinical trial of excellent quality. Among these processes, we highlight the routine checking, calibration and testing of devices used in clinical research.

Finally, we reiterate our understanding of the need for scientific discussion and consider the questions raised to be important. However, our aim was to carry out a randomized clinical trial as close as possible to the clinical reality in Brazil, for the management of signs and symptoms of patients with knee osteoarthritis.

## References

[1] de Paula Gomes CAF, Politti F (2020). de Souza Bacelar Pereira C, et al. Exercise program combined with electrophysical modalities in subjects with knee, all of them with themes related osteoarthritis: a randomised, placebo-controlled clinical trial. BMC Musculoskelet Disord..

[2] Hegedus B, Viharos L, Gervain M (2009). The effect of low-level laser in knee osteoarthritis: a double-blind, randomized, placebo-controlled trial. Photomed Laser Surg..

[3] Coelho Cde F, Leal-Junior EC, Biasotto-Gonzalez DA (2014). Effectiveness of phototherapy incorporated into an exercise program for osteoarthritis of the knee: study protocol for a randomized controlled trial. Trials..

[4] Recommended treatment doses for Low Level Laser Therapy 904 nm wavelength [http://waltza.co.za/wp-content/uploads/2012/08/Dose_table_904nm_for_Low_Level_Laser_Therapy_WALT-2010.pdf]. Accessed 15 Nov 2020.

[5] Stausholm MB, Naterstad IF, Joensen J, Lopes-Martins RAB, Saebo H, Lund H, Fersum KV, Bjordal JM (2019). Efficacy of low-level laser therapy on pain and disability in knee osteoarthritis: Systematic review and meta-analysis of randomised placebo-controlled trials. BMJ Open.

[6] de Paula Gomes CAF, Leal-Junior ECP, Dibai-Filho AV (2018). Incorporation of photobiomodulation therapy into a therapeutic exercise program for knee osteoarthritis: A placebo-controlled, randomized, clinical trial. Lasers Surg Med..

[7] Herpich CM, Leal-Junior ECP, Politti F (2020). Intraoral photobiomodulation diminishes pain and improves functioning in women with temporomandibular disorder: a randomized, sham-controlled, double-blind clinical trial. Lasers Med Sci..

[8] Calamita SAP, Biasotto-Gonzalez DA, De Melo NC (2018). Immediate Effect of Acupuncture on Electromyographic Activity of the Upper Trapezius Muscle and Pain in Patients With Nonspecific Neck Pain: A Randomized, Single-Blinded, Sham-Controlled, Crossover Study. J Manipulative Physiol Ther..

[9] Gomes CAFP, Dibai-Filho AV, Moreira WA (2018). Effect of Adding Interferential Current in an Exercise and Manual Therapy Program for Patients with Unilateral Shoulder Impingement Syndrome: A Randomized Clinical Trial. J Manipulative Physiol Ther..

[10] Gomes CAFP, Dibai-Filho AV, Politti F (2018). Combined Use of Diadynamic Currents and Manual Therapy on Myofascial Trigger Points in Patients with Shoulder Impingement Syndrome: A Randomized Controlled Trial. J Manipulative Physiol Ther..

[11] Herpich CM, Leal-Junior ECP, Gomes CAFP (2018). Immediate and short-term effects of phototherapy on pain, muscle activity, and joint mobility in women with temporomandibular disorder: a randomized, double-blind, placebo-controlled, clinical trial. Disabil Rehabil..

